# Social Calls Produced within and near the Roost in Two Species of Tent-Making Bats, *Dermanura watsoni* and *Ectophylla alba*

**DOI:** 10.1371/journal.pone.0061731

**Published:** 2013-04-24

**Authors:** Erin H. Gillam, Gloriana Chaverri, Karina Montero, Maria Sagot

**Affiliations:** 1 Department of Biological Sciences, North Dakota State University, Fargo, North Dakota, United States of America; 2 Biology Department, Boston University, Boston, Massachusetts, United States of America; 3 Department of Biological Sciences, Texas Tech University, Lubbock, Texas, United States of America; Institut Pluridisciplinaire Hubert Curien, France

## Abstract

Social animals regularly face the problem of relocating conspecifics when separated. Communication is one of the most important mechanisms facilitating group formation and cohesion. Known as contact calls, signals exchanged between conspecifics that permit group maintenance are widespread across many taxa. Foliage-roosting bats are an excellent model system for studying the evolution of contact calling, as there are opportunities to compare closely related species that exhibit major differences in ecology and behavior. Further, foliage-roosting bats rely on relatively ephemeral roosts, which leads to major challenges in maintaining group cohesion. Here, we report findings on the communication signals produced by two tent-making bats, *Dermanura watsoni* and *Ectophylla alba.* We found that both species produced calls in the early morning near the roost that were associated with roostmate recruitment. Calling often ended once other bats arrived at the tent, suggesting that calls may be involved in roostmate recruitment and group formation. The structure and function of these calls are described and future research directions are discussed.

## Introduction

Whether social or solitary in nature, almost all animals associate with one or more conspecifics at some point during their lifetime. Many factors can drive the formation of social groups, such as limited or patchy resource distributions (e.g. [1]), reduced risk of predation (e.g. [2]), enhanced reproductive success (e.g. [3]) and increased information exchange (e.g. [4]).

Regardless of the factors that lead to the formation of social groups, animals regularly face the problem of relocating conspecifics when separated, and communication often plays an important role in facilitating the formation and maintenance of social groups. Such signals involved in group formation, known as “contact calls” [5], are common across many taxa and are often the primary mechanism by which group cohesion is maintained (i.e. [6], [7], [8], [9]).

Bats are an especially interesting group for examining the role of communication in permitting group cohesion, as they are highly social and exhibit extensive diversity in mating systems and social organization across species [10]. Given the social nature of this taxon, it is not surprising that many species produce signals that convey specialized information to receivers. Social calls have been shown to play important roles in the behavior of bats, including offspring recognition [11], mate attraction [12] and advertisement of aggression [13], or distress [14].

Contact calls have been identified in a suite of bat species exhibiting significant variation in ecological and behavioral characteristics. In many species, pups produce unique “isolation calls,” a form of contact calling that allows mothers to relocate offspring after periods of separation [15]. Bechstein’s bats, *Myotis bechsteinii*, are attracted to roosts in which the social calls of conspecifics, but not heterospecifics, are being broadcast [16]. Likewise, greater spear-nosed bats, *Phyllostomus hastatus*, are known to produce group-specific screech calls when exiting a roost; these calls attract group mates to the caller’s location, which presumably facilitates group foraging [17]. Common vampire bats, *Desmodus* rotundus [18], white-winged vampire bats, *Diaemus youngi* [19], [20] and pallid bats, *Antrozous pallidus* [21] have been shown to exchange consistent, individual-specific contact calls that provide information about the location of adult conspecifics.

Contact calling may be especially important for species that exhibit low roost fidelity, either due to the use of roosts that deteriorate quickly or because bats regularly move amongst a set of potential roosts. Under such conditions, individuals must locate roosts and conspecifics by actively searching within their home range, which may be energetically expensive. Spix’s disk-winged bat, *Thyroptera tricolor,* is an especially poignant example of such a behavioral challenge. This species uses highly ephemeral roost sites (furled, tubular leaves) while still maintaining long-term, stable associations with a set of conspecifics [22], [23]. Two of the authors (GC and EHG) have previously documented a contact calling system for this species in which two distinct social calls are exchanged between flying and roosting bats [24] [25]. Flying bats actively searching for a roost produce an ‘inquiry’ call; roosting conspecifics in the area rapidly answer with a ‘response’ call, which is often followed by the flying bat entering the occupied leaf roost. These two social calls have not been documented in any other social contexts, and response calls appear to only be emitted after production of an inquiry call.

The objective of this study was to expand upon previous research on contact calling in *T. tricolor* to other neotropical foliage-roosting bats that face similar challenges in locating conspecifics and maintaining group cohesion. We focus on two species that differ in aspects of their social organization and roosting ecology, in an effort to understand how such behavioural and ecological factors may shape the structure of signalling systems. Due to the paucity of data on the social behaviour and communication systems of tent-making bats, we did not have sufficient background information to form and test specific hypotheses. Instead, this research was aimed at examining the types of acoustic signals involved in social interactions at and near tent roosts, and generating hypotheses about signal function that could be more rigorously tested in future studies. Further, limited behavioural data on social interactions amongst bats, especially those that regularly move between roosts, can be primarily attributed to the difficulty of recording bats within natural roosts. In this study, we attempted to refine field methods for video and audio recording to permit collection of behavioural data under natural, undisturbed conditions in two species that often do not return to the same roost each night.

### Study Species

Thomas’ fruit-eating bat, *Dermanura watsoni,* is a small frugivore found from southern Mexico to northern South America [26]. Amongst tent-making bats, this species modifies the largest number of plants into tent roosts (41 plant species; [27]) and can produce several architectural types of tents. Tents can remain usable for weeks to months, depending upon the plant species. Tent building is believed to be conducted exclusively by males [27]. *D. watsoni* exhibit a mating system of resource-defense polygyny, in which a single male defends an important resource (tent) that attracts females to his location [28]. While multiple *D. watsoni* often share a small home range, roosting groups are not permanent, with females regularly visiting the tents of different males [29]. Both sexes exhibit low roost fidelity, as males are known to intermittently occupy (and recruit females to) several tents within their home range [27].

The Caribbean white tent-making bat, *Ectophylla alba*, is also a small frugivore, and is found exclusively in the Caribbean lowlands of Central America [30]. These bats use eight plant species for tent construction, but most are made from plants in the genus *Heliconia* [31], [32]. Tent lifespan ranges from a few days up to eight weeks [33]. *E. alba* is most commonly found in mixed-sex groups [30], [31] and females have been observed participating in the tent construction process [34]. Caribbean white tent bats maintain long-term associations with specific individuals, and groups have even been documented switching to new tent roosts together [31]. Roost fidelity is also high, with groups having been found to occupy the same tent for up to 45 days [31], [34].

## Methods

Data on *D. watsoni* were collected in January 2011 at two sites within the Golfito Wildlife Refuge (El Naranjal Field Station and La Lechería sector), which is located in southwestern Costa Rica. Data for *E. alba* were collected in March 2011 at Refugio de Mariposas and Tapiria National Refuge, both located in Sarapiquí, central Costa Rica. All described work was approved by the Institutional Animal Care and Use Committee of North Dakota State University (Protocol # A110017) and the Costa Rican government (permit # 002-2011-SINAC). During the sampling periods for both species, reproductive females were pregnant.

At all sites, we located tent roosts during the day and then monitored the roosts at night using video and audio equipment. We located tents for observation using two different methods. At La Lechería and Saripiquí, we searched the forest during the day for occupied tents, taking care to avoid disturbing the bats in the roost. At El Naranjal (*D. watsoni* only), we also searched for tents during the day, but once an occupied roost was located, all individuals were captured using a modified hoop net. For each captured bat, standard measurements were taken, including sex, age, reproductive condition, mass, and forearm length, and a uniquely numbered metal ring (Porzana Ltd, UK) were attached to the forearm. In addition, radio transmitters (Model LB-2N, 0.35 grams, Holohil Systems, Canada) were attached to six adult males. Roosts were subsequently located by tracking bats to their tents every day until the radio transmitters fell off or the batteries died (∼10 days).

For all sites and species, one to two tent roosts were selected each evening for monitoring. After all bats had emerged from the roost, a video camera (Sony CCD-TRV138, NY, USA) and infrared light source (IRLamp6, Wildlife Engineering, Lacrosse, WI, USA) was placed underneath the tent and oriented toward the roosting area. In addition, a multi-microphone ultrasonic recording system (Avisoft UltrasoundGate 416 with four CM16 microphones, Avisoft Bioacoustics, Berlin, Germany) was deployed, with one microphone mounted next to the video system and the other three microphones placed at different locations within 10 m of the tent. The video system and a single microphone monitored activity within the roost, while the remaining microphones detected any calls produced by bats flying in the vicinity of the tent. We initially recorded continuously throughout the night until bats returned to the roost in the early morning, but repeated observations revealed that bats were only active at the tent near dawn. Hence, later recordings were begun at 03∶00 and continued until bats returned to the tent at dawn (∼ 04∶45–06∶00). If no bats occupied the tent by 06∶30, we concluded that individuals had selected a different day roost, and recordings were terminated.

At El Naranjal, a researcher remained near the roost throughout the recording period to monitor for the presence of radiotagged bats using a telemetry receiver (TRX-1000S, Wildlife Materials Inc., Carbondale, Illinois) and three-element Yagi antenna. When a strong signal was detected, potentially indicating that a tagged bat had returned to the roost, the researcher checked the screen of the video camera to confirm the presence of the radiotagged individual in the tent. If present, the identity of the bat and time of return was logged; this information was then used to identify individuals during subsequent video analysis. At La Lechería and Saripiquí, information about monitored bats was not known beforehand, so after recording was complete, we attempted to capture bats for further inspection and gathering of standard measurements. It was not feasible to capture bats before recording, as this disturbance would cause the group to abandon the roost. When possible, we used this post-recording capture information to identify the sex of the bat(s) filmed within the roost. Under some conditions, such as when a roost contained one male and one female, we could not definitively assign sex to the bats recorded on the video, hence sex information was not used in analyses.

Video data were analyzed using Final Cut Pro 7 (Apple, Inc, USA), while audio data was analyzed using Avisoft SasLab Pro. For each night, we determined the number of bats present at the roost. Where possible, data from monitoring of radio tags or post-recording captures were used to assess the sex of the individuals in the roost. We conducted a Mann-Whitney U non-parametric test to asses if arrival time at the roost differed significantly between males and females. Audio and video data were aligned, and recorded calls were matched to specific individuals on the accompanying video recording, when possible. In all but one case, this was possible because only a single male was in the tent. In the one instance in which we identified the female as the caller, the social calls were produced as the bat was entering the roost, which was associated with changing amplitude of the recorded calls. For each high-quality call, we assessed a suite of call measurements, including duration (Dur), inter-call interval (ICI), start frequency (F_start_), end frequency (F_end_), and peak frequency (F_peak_), which corresponds to the frequency of maximum energy in the call. We also assessed the peak frequency of the fundamental signal (F_fund_) and the first four harmonics (F_H1_−F_H4_). All call variables were collected using the Automatic Parameter Measurements function in SasLab Pro, although each call was manually inspected to ensure that measurements were taken appropriately (i.e. correct harmonic, noise not included).

## Results

Video and audio sampling was conducted for *D. watsoni* on eight nights at eight tents across the two study locations (8 groups of bats with some overlap in group composition). We were able to identify the sex of all recorded *D. watsoni*, which included 6 males and 9 females. There was a trend for males to return to the roost earlier (05∶08–05∶27) compared to females (05∶24–05∶56), although this difference was not statistically significant (Mann-Whitney U test: P = 0.15), likely due to small sample size. Among the eight tents sampled, we only ever observed a single male in each tent; males were either alone or with one or more females.

**Figure 1:**
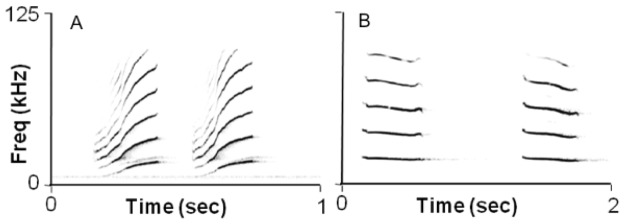
Described social calls. Sonogram of a typical social call produced within or near the roost by A) *D. watsoni* and B) *E. alba.*

We recorded one distinctive type of social call (Figure 1a; Table 1) that was produced within the roost by *D. watsoni*. In total, we recorded 873 social calls from 4 individuals (3 males, 1 female), although 97% (846 calls) came from one night in which we sampled two tents ∼6 m apart that were both occupied by single males. In this case, males appeared to produce calls in a reactive manner, with a back-and-forth pattern of call emission between the two individuals. Of the remaining recorded calls, only two were emitted by a female, and all recorded calls were emitted by bats within the roost. Temporal patterns of call production were not uniform, with signals sometimes emitted singly, while other times a rapid bout of two or more calls was produced. Specifically, 23% of calls were emitted as a single, stand-alone call, while the remaining signals were emitted as a bout with very short intervals between each call (37% as a pair 27% as a triplet, and 13% as a group of four or more calls). On 6 January 2011, we observed a male arrive at a tent and produce a series of 15 social calls (Figure 1a) over a 2 min period, after which a female entered the roost.

**Table 1:** Average parameter measurements for the social calls recorded at or near the roost for *D. watsoni* and *E. alba.*

	Dur	F_start_	F_end_	F_peak_	F_fund_	F_H1_	F_H2_	F_H3_	F_H4_
***D. watsoni***	202.91	8.46	17.55	31.35	17.70	33.08	48.28	65.37	80.35
***E. alba***	37.08	23.10	18.18	19.03	19.03	37.81	56.13	75.07	NA

Duration is reported in ms and all spectral variables are reported in kHz. No value is reported for F_H4_ in *E. alba*, as a fourth harmonic was not detected in any calls of this species.

We sampled *E. alba* on four nights, which included recording of three different social groups. Bats returned to the roost singly from 04∶45–05∶56. One roosting group was composed of a single female and three males; the other two roosting groups were not successfully captured, as they did not return to the same tent on the night of recording. Sex information for the one group could not be used to determine sex-related differences in return times, as we could not definitively identify individuals in the video data. We found that one distinctive type of social call (Figure 1b; Table 1) was produced by *E. alba* in the vicinity of a tent, with a total of 50 calls identified in our recordings. Unlike *D. watsoni*, we found that social calls were only produced by bats flying near the tent. On one occasion (12 March), we observed that a pair of social calls was produced immediately before a flying bat entered a roost that was already occupied by another individual, but we could not determine if the social calls were produced by the flying or roosting individual. Calls were primarily emitted in pairs (80%), with three incidences of triplets (8%) and only one call emitted singly (2%).

## Discussion

Results from our work on *D. watsoni* led to two noteworthy findings. First, bats occupying a tent roost produce a distinctive multi-harmonic social call (Figure 1a). Further, almost all social calls were produced by males, with only one instance of a female emitting the described social call. Second, calls were exclusively recorded in the 60 min prior to sunrise. For nights that we recorded from sunset to sunrise, no bat activity was detected on the video or audio recordings until the very early morning hours.

We observed two different behavioral outcomes (other than no response) to the production of social calls by male *D. watsoni*. In one instance, call production by a male was quickly followed by a nearby female entering the roost. Since this is a single observation, we cannot make any definitive conclusions, yet it presents the possibility that social calls may play a role in male recruitment of females. Second, calling could be involved in establishing territory boundaries between males in nearby roosts. On the night of 8 January 2011, from which we recorded the greatest number of social calls, video and audio data were collected separately at two roosts that were within 10 m of each other, both of which were occupied by a male *D. watsoni*. Calling behavior of each bat appeared to be primarily triggered by production of social calls from the other male, leading to a back-and-forth between the two bats. In this case, neither bat successfully recruited a female to the roost. While more data are needed, these data suggest that a future hypothesis worthy of testing would address a potential territorial defense function of this signal. Social calls play an important role in establishing territorial boundaries in a diverse suite of taxa, including mammals [35], [36], birds [37], [38] and amphibians [39], among others.

We found that *E. alba* also produced a distinctive type of social call at the roost, although unlike *D. watsoni*, the calls were only recorded from individuals flying in the vicinity of a tent. We did not observe the behavioral sequence “flying bat calls → roosting bat calls → flying bat enters occupied roost”, although our limited sample size could have meant that we simply missed this behavior. Due to the small number of bats sampled here, it is difficult to draw further conclusions about the behavioral function of these calls.

Our findings suggest that both study species exhibit an active social calling system that appears to facilitate interactions between group mates, either while flying (*E. alba*) or roosting (*D. watsoni*). *D. watsoni* are known to only form groups with a single male, while *E. alba* form mixed sex groups in which more than one male is sometimes present. Our observation that social calls are mainly produced by roosting male *D. watsoni* fits with the resource-defense polygynous mating system of this species. Specifically, males attempt to recruit females to a resource they control (a tent) to gain access to mating opportunities [40]. Thus, it would be anticipated that males in a tent produce calls, while females in a tent would not need to call, as they have already located the desired resource. Despite this prediction, we did observe a female *D. watsoni* produce two social calls, suggesting that in some behavioral contexts producing the described signal may be advantageous to females.

Unlike *D. watsoni*, female *E. alba* are known to participate in the tent-making process [34]), suggesting that females may aggregate at tents for reasons other than access to a male-controlled resource. Further, stable groups exhibit high roost fidelity, suggesting that returning to the same location repeatedly may generally be an effective method for maintaining group cohesion. Under such conditions, we would predict that both sexes would call when occupying a roost to attract other individuals and gain benefits of group formation, such as thermoregulation and predator avoidance. Our observation that most social calls in *E. alba* are produced on the wing rather than in the roost may suggest that in this species, group formation is more important than attempting to attract others to a specific roost site. While our data did not allow us to determine if both sexes were producing social calls in *E. alba*, we would predict that this is the case, and encourage future data collection to assess this hypothesis.

Our results suggest that species using ephemeral roosting resources may use acoustic communication systems for interacting with conspecifics, whether it be for maintaining group cohesion, attracting mates, or establishing territorial boundaries. While our data do not permit us to reach conclusive findings, these results have allowed us to generate hypotheses about signal function that can be more rigorously tested in future research involving call playbacks and characterization of individual responses to social calls. More extensive observations on the two study species, plus additional leaf-roosting bats, would also be valuable for understanding how ecological, behavioral, and evolutionary forces have shaped the characteristics of such signaling systems.
